# Comparative genomic analyses reveal genetic characteristics and pathogenic factors of *Bacillus pumilus* HM-7

**DOI:** 10.3389/fmicb.2022.1008648

**Published:** 2022-11-07

**Authors:** Qian Wang, Lei Zhang, Yiju Zhang, Huamin Chen, Jianghua Song, Mingjie Lyu, Rui Chen, Lixin Zhang

**Affiliations:** ^1^Anhui Province Key Laboratory of Integrated Pest Management on Crops, College of Plant Protection, Anhui Agricultural University, Hefei, China; ^2^Institute of Crop Germplasm and Biotechnology, Tianjin Academy of Agricultural Sciences, Tianjin, China; ^3^State Key Laboratory for Biology of Plant Disease and Insect Pests, Institute of Plant Protection, Chinese Academy of Agricultural Sciences, Beijing, China; ^4^College of Horticulture, Anhui Agricultural University, Hefei, China

**Keywords:** *Bacillus pumilus*, complete genome sequence, pan-core genome, comparative genomic analysis, pathogenic gene

## Abstract

*Bacillus pumilus* plays an important role in industrial application and biocontrol activities, as well as causing humans and plants disease, leading to economic losses and biosafety concerns. However, until now, the pathogenesis and underlying mechanisms of *B. pumilus* strains remain unclear. In our previous study, one representative isolate of *B. pumilus* named HM-7 has been recovered and proved to be the causal agent of fruit rot on muskmelon (*Cucumis melo*). Herein, we present a complete and annotated genome sequence of HM-7 that contains 4,111 coding genes in a single 3,951,520 bp chromosome with 41.04% GC content. A total of 3,481 genes were functionally annotated with the GO, COG, and KEGG databases. Pan-core genome analysis of HM-7 and 20 representative *B. pumilus* strains, as well as six closely related *Bacillus* species, discovered 740 core genes and 15,205 genes in the pan-genome of 21 *B. pumilus* strains, in which 485 specific-genes were identified in HM-7 genome. The average nucleotide identity (ANI), and whole-genome-based phylogenetic analysis revealed that HM-7 was most closely related to the C4, GR8, MTCC-B6033, TUAT1 and SH-B11 strains, but evolutionarily distinct from other strains in *B. pumilus*. Collinearity analysis of the six similar *B. pumilus* strains showed high levels of synteny but also several divergent regions for each strains. In the HM-7 genome, we identified 484 genes in the carbohydrate-active enzymes (CAZyme) class, 650 genes encoding virulence factors, and 1,115 genes associated with pathogen-host interactions. Moreover, three HM-7-specific regions were determined, which contained 424 protein-coding genes. Further investigation of these genes showed that 19 pathogenesis-related genes were mainly associated with flagella formation and secretion of toxic products, which might be involved in the virulence of strain HM-7. Our results provided detailed genomic and taxonomic information for the HM-7 strain, and discovered its potential pathogenic mechanism, which lay a foundation for developing effective prevention and control strategies against this pathogen in the future.

## Introduction

*Bacillus pumilus*, an endospore-forming Gram-positive bacterium, residing in stratospheric air, soil, deep-sea sediments, and some extreme environments, is one of the best-known *Bacillus* species (Logan et al., 2009; Stepanov et al., 2016; Zhou et al., 2022). *B. pumilus* plays a crucial role in industry for producing abundant extracellular enzymes and secondary metabolites, and shows high adaptability and stress resistance (Bonifer et al., 2019; Zuo et al., 2022). This species is commonly utilized as probiotics in animal (Sanders et al., 2003; Hong et al., 2005). *B. pumilus* has also been widely used in agriculture for their beneficial actions on plants, such as plant-growth-promoting effect by facilitating plant nutrient uptake, phytohormone synthesis, phosphate solubilization, and biological nitrogen fixation (Hernandez et al., 2009), as well as antagonistic activities by producing antimicrobial agents (Huang et al., 1992; Agarwal et al., 2017; Dai et al., 2021). Meanwhile, increasing research has revealed that some *B. pumilus* strains were determined as pathogenic due to causing diseases in human and plants. For instance, foodborne illness (Logan, 2012) and cutaneous infection (Tena et al., 2007; Johnson et al., 2008) in human was associated with toxicity of *B. pumilus* strains. Several previous reports confirmed that *B. pumilus* caused diseases in a variety of forest trees (Kovaleva et al., 2015; Mazlan et al., 2019), fruits (Saleh et al., 1997; Galal et al., 2006; Li et al., 2009; Song et al., 2018), and vegetables (Peng et al., 2013), including the staple-food potato (Bathily et al., 2010), resulting in enormous economic losses and potential biological risk. However, to date, the pathogenic mechanism of these *B. pumilus* strains remain unclear.

Advances in sequencing technologies and the rapid development of genomic tools facilitate researchers to gain essential insights into the molecular basis of the strains at the genome level. So far, 177 genome assemblies of *B. pumilus* strains have been deposited into the National Center for Biotechnology Information (NCBI) public database, including 15 complete genomes, 4 chromosomes, and 158 draft genomes, most of which have been sequenced in the past 3 years. Comparative genomic approaches have been proven to be extremely valuable for functional characterization and classification of bacteria and fungi (Jothi et al., 2006). Recently, non-pathogenic *B. pumilus* have been investigated for their genomic features, phylogenetic relationships and evolutionary mechanisms by genomic analysis. Comparative genomic analysis of *B. pumilus* strains 7P and 3–19, revealed that nucleotide variants affected the streptomycin resistance and overproduction of extracellular hydrolases in *B. pumilus* 3–19 (Pudova et al., 2022). The niche-specific differences in genome expansion of antibacterial *B. pumilus* SF-4 and other eleven strains were revealed by genome mining and comparative genome analysis (Iqbal et al., 2021). Comparative analysis of marine-derived and the terrestrial *B. pumilus* strains revealed the evolutionary relationships, differentiation, and environmental adaptation (Fu et al., 2021). Yuan and Gao (2015) conducted genomic analysis of the ginger pathogen *B. pumilus* strain GR8, which revealed plant candidate pathogenic genes. Taken together, previous reports focused on the non-pathogenic strains of *B. pumilus*, whereas the pathogenic strains of *B. pumilus* and the underlying pathogenic mechanisms are remain elusive.

In our previous work, we isolated the pathogenic strain HM-7 from symptomatic fruit, which causes bacterial soft rot in melon. This strain was recognized as *B. pumilus* by sequence analysis of the 16S rRNA and the b-subunit of DNA gyrase (*gyrB*) gene (Song et al., 2018). In this study, we sequenced and assembled the genome of *B. pumilus* HM-7 and carried out comparative genomic analysis based on pan-core genome analysis, average nucleotide identity, phylogenetic and genomic collinearity analysis of all the available complete genomes or chromosomes of *B. pumilus* strains, as well as six closely related species (*Bacillus anthracis*, *Bacillus cereus*, *Bacillus licheniformis*, *Bacillus safensis*, *Bacillus subtilis*, and *Bacillus thuringiensis*). Genome annotation and analysis of pathogenic-associated genes revealed the genome characteristics, phylogenetic and taxonomic status of HM-7. Our results provide new insights into the pathogenic properties of *B. pumilus* and lay a solid foundation for controlling bacterial diseases caused by *B. pumilus* HM-7.

## Materials and methods

### The bacterial strain and DNA extraction

The representative isolate *B. pumilus* HM-7 used in this study was previously recovered from diseased tissues of muskmelon fruit (cv. “Xingtian20”) in Huainan, Anhui Province, China. The strain HM-7 was shake-cultured (220 rpm) in Nutrient Agar (NA) liquid medium (0.3% beef extract, 1.0% glucose, 0.5% peptone, 0.05% yeast extract) at 28°C for 24 h, and bacterial cells were harvested by centrifugation at 5,000 × g for 10 min at 4°C. Genomic DNA was extracted from the bacterial pellet using the TAKARA Bacterial DNA Kit (TaKaRa, Japan) according to the manufacturer’s instructions. The concentration and quality of genomic DNA was determined by NanoDrop (Thermo Fisher Scientific, Loughborough, UK) and agarose gel electrophoresis. *B. pumilus* HM-7 was preserved in the China Center for Type Culture Collection as CCTCC AB 2019388.

### Genome sequencing, assembly and annotation

The whole genome sequencing of *B. pumilus* HM-7 was performed using the PacBio RS II single-molecule real-time (SMRT) sequencing platform. *De novo* assembly of high-quality long reads was carried out using HGAP (Chin et al., 2013) and further polished by Quiver (Chin et al., 2013). The resulting sequence data was assembled into a single contiguous genome. Genome annotation was performed using Prokka version 1.14 (Seemann, 2014). Open reading frames (ORFs) were predicted by Prodigal (Hyatt et al., 2010). Ribosomal RNA (rRNA) genes and transfer RNA (tRNA) genes was identified by the RNAmmer V1.2 (Lagesen et al., 2007) and tRNAscan-SE V2.0 (Lowe and Eddy, 1997), respectively. The interspersed repetitive sequences and tandem repeats were detected by RepeatMasker V4.1 (Chen, 2004) and the Tandem Repeats Finder (Benson, 1999). Genome visualization was carried out using the CGView Comparison Tool (Grant et al., 2012).

Functional analysis of the protein-coding genes was performed using Clusters of Orthologous Groups (COG) (Tatusov et al., 1997), Gene Ontology (GO) (Levasseur et al., 2013; Jones et al., 2014), and Kyoto Encyclopedia of Genes and Genomes (KEGG) (Kanehisa et al., 2016) databases. Pathogenicity analysis was conducted via a whole genome Blast search of the Carbohydrate-Active enZYmes (CAZy) database (Levasseur et al., 2013), the Virulence Factors Database (VFDB) (Chen et al., 2012), and the Pathogen Host Interactions (PHI) database (Winnenburg, 2006). An E-value cut-off of 1*e*-10 was set for the BLAST analysis.

### Pan-genome analysis

A total of 20 complete genomes or chromosomes of *B. pumilus* strains and six type strains of closely related species were retrieved from the NCBI ftp site^1^ and listed in Supplementary Table 1. All genomes were re-annotated using Prokka (version 1.14) (Seemann, 2014) with identical default parameters. The pan-genome analysis of these genomes was evaluated, using the Roary program (Page et al., 2015) with a blast identity cutoff of 97% for comparison between *B. pumilus* strains, and a 40% cutoff for comparison between *Bacillus* species. Furthermore, we used R to map the petal plot to visualize the number of unique genes in each strain derived from the pan-genome analysis, as well as an UpSet plot to visualize the intersecting gene sets between *B. pumilus* HM-7 and five closely *B. pumilus* strains.

### Average nucleotide identity and phylogenetic analysis

ANI were calculated by using JSpecies software (Richter and Rosselló-Móra, 2009) with default parameters to elucidate the interspecific relationship of these strains. A Pearson correlation matrix was generated, and correlation analysis ordered by hierarchical clustering was performed according to the procedures of Espariz et al. (2016).

Gene clusters that were shared among all strains and contained only single gene copies from each strain were referred to as single-copy genes (Zhong et al., 2018). We constructed phylogenetic tree based on single copies from the clustering result of OrthoFinder version 2.5 (Emms and Kelly, 2015) with default parameters. These protein sequences of each strains obtained from Prokka were selected and rooted using MUSCLE V3.8 (Edgar, 2004) with default parameters to perform multiple sequence alignment. The alignment was trimmed with GBlocks 0.91b (Gblocks $i -b4 = 5 -b5 = h -t = p -e = 0.2) (Talavera and Castresana, 2007) and used to infer the evolutionary history of strains with Randomized Axelerated Maximum Likelihood Algorithm (RAxML) (Stamatakis et al., 2008), based on the GAMMAJTT model for proteins (raxmlHPC-PTHREADS-SSE3 -T 64 -f a -x 123 -p 123 -N 1000 -m PROTGAMMAJTT -k -O -n $output_tag.tre -s $input_tag.phy). The reliability of the inferred tree was tested by bootstrapping with 1,000 replicates. The online tool iTOL^2^ was used for visualization.

### Genomic collinearity analysis and three HM-7-specific regions

We analyzed the genomic architectures and syntenic relationships among the genomes clustered together with *B. pumilus* HM-7 using the Mauve Alignment System (Darling et al., 2004). Genomic islands (Gls) were determined with the IslandViewer 4 using four independent methods IslandPick, SIGI-HMM, IslandPath-DIMOB, and Islander. Prophages were detected using the PHASTER (PHAge Search Tool Enhanced Release).^3^ Clustered Regularly Interspaced Short Palindromic Repeats (CRISPR) elements were identified using CRISPR Finder software (Grissa et al., 2007). The output file “gene_presence_absence.csv” (Supplementary Table 2) was analyzed using Microsoft Excel to identify strain-specific genes present in HM-7 and absent in other strains based on the results of Roary analysis.

## Results

### Genomic features and annotation of *B. pumilus* HM-7

**FIGURE 1:**
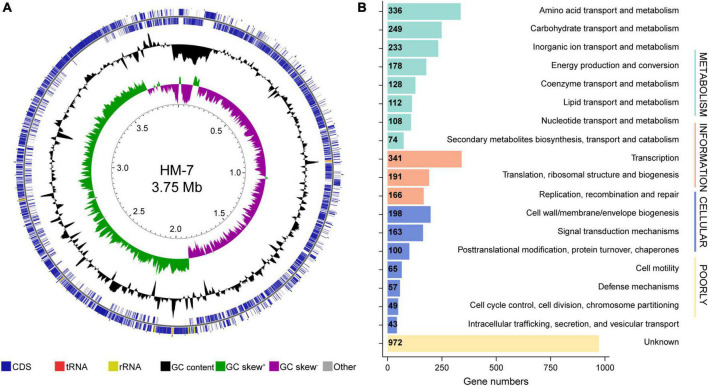
Genome analysis of *B. pumilus* strain HM-7. **(A)** From the inner to the outer circle: Circle 1, genomic position; Circle 2, GC skew; Circle 3, GC content; Circle 4 and 5, predicted protein-coding sequences (CDS) transcribed anticlockwise (inner part) and clockwise (outer part), respectively. **(B)** COG function classification of genes in HM-7, grouped into four main parts: Metabolism, cellular processes, information, and poorly.

In order to obtain a high-quality genome sequence of *B. pumilus* HM-7, 5.27 Gb raw reads were produced from the PacBio platform, with an N50 length of 13,912 bp and an average length of 8,640 bp, accounting for approximately 1,340-fold genome coverage. *De novo* assembly of high-quality long reads generated a single contiguous sequence (contig) with a size of 3,951,520 bp and 41.04% GC content (Table 1). In total, 4,111 genes, three sets of 16S, 23S, and 5S rRNA operons, 24 rRNA genes, 82 tRNA genes, and 1 tmRNA were identified. Moreover, 136 interspersed repetitive sequences (IRSs) and 118 tandem repeats (TRs) were also discovered in this assembled genome (Table 1). The genomic loci of these protein-coding, rRNA, and tRNA genes were unevenly distributed across the genome (Figure 1A).

**TABLE 1:** General features of the *B. pumilus* HM-7 genome.

Genome	*B. pumilus* HM-7
Size	3,951,520 bp
GC content	41.04%
Number of coding sequences	4,111
Number of tRNA	82
5s rRNA number	8
16s rRNA number	8
23s rRNA number	8
TmRNA	1

To further determine the functions of the 4,111 annotated genes, we performed BLAST searches against the COG, GO, and KEGG databases. Of these, 3,479 (84.6%) predicted genes were assigned to the COG categories (Figure 1B): 1,418 of the genes were related to metabolism, 675 to cellular processes, 698 genes to information, and 972 genes to poorly characterized. A total of 919 (22.4%) genes could be assigned with certain GO definitions, including 677 genes in biological processes, 577 genes in cellular components, and 654 genes in molecular functions. Enriched GO terms focused on cellular process (608), metabolic process (528), cellular anatomical entity (567), obsolete cell part (534), and catalytic activity (454) (Supplementary Figure 1A). The KEGG annotation led to the identification of 1,268 genes with definite functions. Among the categories, metabolism was the largest group, containing metabolic pathway (613 genes), biosynthesis of secondary metabolites (294 genes), microbial metabolism in diverse environments (187 genes), biosynthesis of amino acids (131 genes), and others. The second largest group was the environmental information processing, mainly composed of ABC transporters (155 genes) and two-component system (109 genes) (Supplementary Figure 1B).

### Potential pathogenesis-related genes in HM-7 genome

**FIGURE 2:**
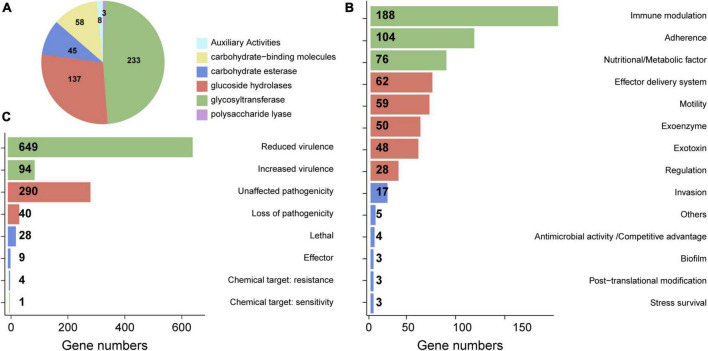
The annotation of CAZymes, VFDB, and PHI in *B. pumilus* HM-7 genome. **(A)** Distribution of CAZymes categories and gene number. **(B)** The functional categories of the *B. pumilus* HM-7 virulence genes according to the classification in the VFDB. **(C)** PHI classification and the genes number in HM-7. The list of genes are presented in Supplementary Tables 3–5.

To investigate the pathogenic mechanisms of *B. pumilus* HM-7, we carried out the homology screening of potential virulence factors based on the CAZymes, VFDB, and PHI databases. CAZymes is an important virulence factors that involved in metabolism of host cell wall and required for invasion into host tissue (Lee et al., 2014). As a result, 484 (11.77%) CAZymes genes were identified in HM-7 genome, which were classified into six classes, encompassing auxiliary activities (AAs), glycosyltransferase (GT), glycoside hydrolases (GHs), carbohydrate-binding molecules (CBMs), carbohydrate esterases (CEs), and polysaccharide lyases (PLs). Of these, the most abundant enzymatic family was GT with 233 CAZyme encoding genes that could be further divided into 19 different families (Supplementary Table 3). The second most frequent enzyme subfamily was GHs (137 genes), followed by CBMs (58 genes), CEs (45 genes), AAs (8 genes), and PLs (3 genes) (Figure 2A and Supplementary Table 3).

The VFDB provides up-to-date knowledge of the virulence factors (VFs) of various bacterial pathogens (Yao et al., 2020). As a result, 650 (15.81%) virulence genes were identified, which could be assigned to 14 basal categories of VFDB. The largest identified category was immune modulation (188 genes), which facilitates the survival of pathogenic bacteria by controlling the host immune system, including anti-phagocytosis, disruption, and depletion of the complement system (Liu et al., 2022). *pdgA*, the most abundant gene in HM-7 genome, is involved in PG N-deacetylation to evade the host innate immune system (Supplementary Table 4; Coullon et al., 2020). Adherence, as the primary step in bacterial pathogenesis (Hori and Matsumoto, 2010), was detected as the second category, and consisted of 104 genes, such as chaperonin GroEL (*groEL*), immunogenic lipoprotein (*IlpA*), pili (*pilD*, *pilJ*, *pilR*), and EF-Tu (*tuf*) (Figure 2B and Supplementary Table 4). A previous study indicated that GroEL, pili, and EF-Tu may facilitate the invasion of the pathogenic *B. pumilus* strain GR-8 through motility and adhesion (Yuan and Gao, 2015). This suggests that these genes might play crucial roles for the pathogenesis of *B. pumilus* HM-7. In addition, distributions and abundances of nutritional/metabolic factors (metal uptake and metabolic adaptation), effector delivery systems (e.g., type II/III/IV/VII secretion system), motility, exoenzyme, exotoxins, and other categories were also identified in the genome of HM-7 (Figure 2B and Supplementary Table 4).

The PHI database contains experimentally verified pathogenicity, virulence, and effector genes from bacterial, fungal and protist pathogens (Urban et al., 2020). We predicted a total of 1,115 (27.12%) PHI genes, which were classified into five categories: virulence, pathogenicity, chemical susceptibility, effector, and lethal. Most of the PHI genes were assigned to the “virulence” class (743 genes), followed by the “pathogenicity” class (330 genes). Furthermore, 28, 9, and 5 genes were annotated as “lethal,” “effector,” and “chemical target” (Figure 2C and Supplementary Table 5). Taken together, these pathogenesis-related candidate genes offered important clues for understanding the pathogenic mechanisms of *B. pumilus* HM-7.

### Pan-core genome analysis

To comprehensively determine the diversity and strain-specific characteristics among *Bacillus* species, pan-core analysis was performed using 21 strains of *B. pumilus* and six closely related *Bacillus* species. As expected, evidence from phylogenetic tree and core-pan gene numbers indicated that the percentage of unique genes was divergent in 27 strains of *Bacillus* spp. (Supplementary Figure 2A). The number of unique genes in *B. subtilis* 168 (1,002 genes), *B. licheniformis* SCDB-14 (1,226 genes), *B. thuringiensis* ATCC-10792 (1,231 genes), *B. anthracis* Ames-Ancestor-A2084 (877 genes), and *B. cereus* BC33 (466 genes) strains was much higher than those in the *B. pumilus* strains (Supplementary Figure 2B).

**FIGURE 3:**
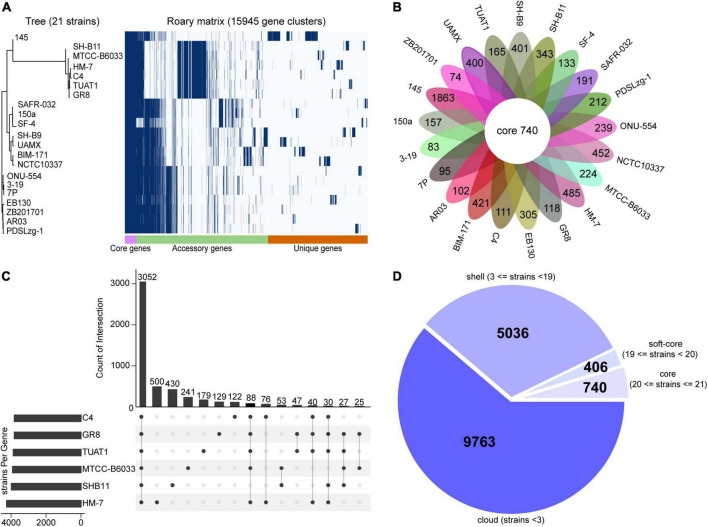
Pan-core genome analysis of 21 strains of *B. pumilus*. **(A)** Gene distribution of 21 *B. pumilus* strains based on the gene presence–absence matrix generated from Roary. A purple box, a green box, and an orange box to represent the core gene, accessory genes and specific genes, respectively. The phylogenetic tree on the left represents the phylogenetic relationships among the strains involved in the corresponding pan-genome. **(B)** Flower petal plot of 21 strains. Pan-genome analysis of 21 strains yielded unique genes for each strain, the numbers of which are shown on each petal plot. The center Circos shows that the number of core gene obtained from 21 strains with hard core threshold. **(C)** Upset plot show the gene set derived from the six strains. Bar numbers are sorted in descending order. Each bar represents the corresponding overlap core genes between two strains. Information provided on the left represent corresponding strain set. **(D)** Pie plot of 21 strains. Pan-genome analysis of 21 strains yielded accessory genes, including shell and cloud genes.

In terms of 21 strains of *B. pumilus*, five strains including MTCC-B6033, TUAT1, C4, SH-B11, and GR8 showed closer relationships to HM-7, which were clustered in the same branch (Figure 3A). The pangenome comprised 15,945 genes, and 740 genes shared by all *B. pumilus* strains (Figure 3B). The 8,631 genes present in more than one strain formed the accessory genome, and 6,574 genes specific to one single strain formed the unique genome ranging from 83 (*B. pumilus* 3–19) to 1,863 (*B. pumilus* 145) genes (Figures 3A,B). *B. pumilus* strain 145 also contained the highest percentage of unique genes (192 genes) within the *Bacillus* species (Supplementary Figure 2B). This was consistent with the previous study that strain 145 was evolutionarily distant from the other *B. pumilus* strains (Figure 3A; Iqbal et al., 2021). The soft core, shell, and cloud contained 406, 5,036, and 9,763 genes, respectively (Figure 3D).

Further analysis of the six closely related *B. pumilus* strains led to the identification of 3,052 core genes, 1,601 unique genes, and 917 accessory genes (Figure 3C and Supplementary Table 2). Notably, HM-7 possessed the most abundant unique genes (500), which was more than the other five strains (Figure 3C).

### Average nucleotide identity and phylogenetic analysis

**FIGURE 4:**
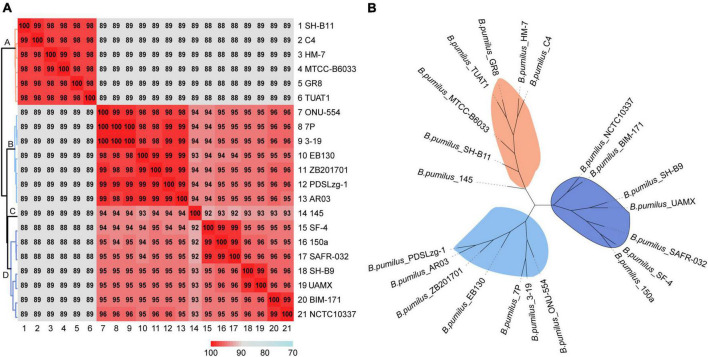
Evolutionary relationships of 21 strains of *B. pumilus*. **(A)** Heatmap and dendrogram of average nucleotide identity among different strains. **(B)** Phylogenetic tree based on total single-copy orthologous.

To confirm the taxonomic identity and explore the phylogenetic relationship of these *B. pumilus* strains, pairwise ANI values were calculated, and a phylogenetic tree was constructed based on single-copies shared by all genomes. As a result, MTCC-B6033, TUAT1, C4, SH-B11, GR8, and HM-7 were clustered together, sharing more than 98% ANI with each other and 89% ANI with the other 15 strains of *B. pumilus* (Figure 4A). According to the ANI matrix and phylogenetic tree, 21 strains of *B. pumilus* were classified into four distinct clades (Figures 4A,B). HM-7 was clustered with MTCC-B6033, TUAT1, C4, SH-B11, and GR-8 (Figures 4A,B). Clade B comprised of seven strains: including ONU-554, 7P, 3-19, EB130, ZB201701, PDSLzg-1, and AR03. Clade D comprised of the other seven strains: SF-4, 150a, SAFR-032, SH-B9, UAMX, BIM-171, and NCTC10337 (Figure 4A). However, *B. pumilus* 145 was evolutionarily distant from the other strains and regarded as a separate clade C (Figure 4A and Supplementary Figure 3A). The ANI matrix and phylogenetic tree of all 27 *Bacillus* species supported this classification, in which *B. pumilus* 145 was obviously divergent from other *B. pumilus* strains (Supplementary Figure 3).

In addition, these 27 *Bacillus* strains were clearly clustered into five clades (Supplementary Figure 3A). *B. safensis* PgKB20 shared more than 89% ANI with 21 strains of *B. pumilus*, and 72–73% with other strains of *Bacillus* species (Supplementary Figure 3A). *B. safensis* PgKB20 was also clustered together with *B. pumilus* strains, but clearly separated from other *Bacillus* species (*B. anthracis* Ames-Ancestor-A2084, *B. cereus* BC33, *B. licheniformis* SCDB-14, *B. subtilis* 168, and *B. thuringiensis* ATCC-10792) (Supplementary Figure 3B). These results suggested that *B. safensis* PgKB20 was more closely related to *B. pumilus* than the other five strains of *Bacillus* species, confirming previous observations (Fu et al., 2021).

### Genomic collinearity analysis and specific-regions of HM-7

**FIGURE 5:**
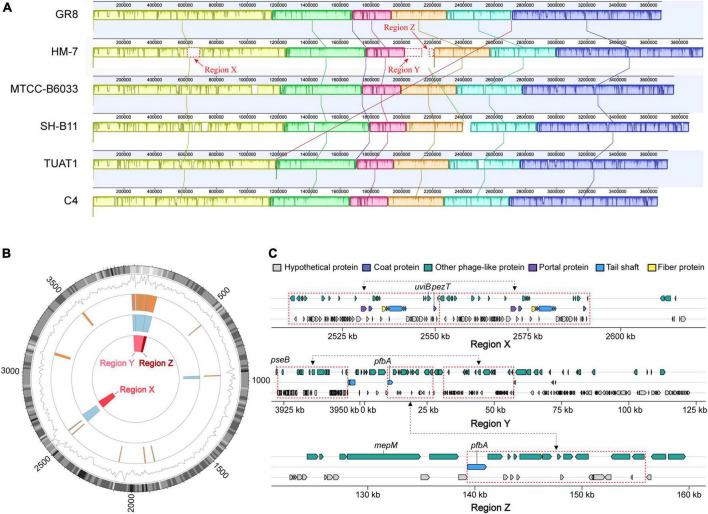
Comparative analysis and mobile genetic elements in the *B. pumilus* strains. **(A)** MAUVE alignment of the genome sequences of *B. pumilus* MTCC-B6033, TUAT1, C4, SH-B11, HM-7 and GR-8. Boxes in the same color represent homologous regions [local colinear blocks (LCBs)] between *B. pumilus* genomes. Uncolored regions within the LCBs or in-between LCBs indicate the presence of specific sequences in that strain. **(B)** Circular genome maps showing the locations of mobile genetic elements and three unique genomic regions (X, Y, Z) in *B. pumilus* strain HM-7. From the inner to the outer circle: Three unique genomic regions position (red boxes); prophages position (light blue boxes); GEIs position (orange boxes); black lines and gray heatmap indicate gene density and GC content, respectively. **(C)** Detailed information and predicted functions of genes located in the regions of X, Y, Z. Important virulence factors were marked with gene ID. Three pairs of blocks (dashed red) linked with black arrows represent repeat fragments.

Collinearity analysis can further identify the uniformity and variability among bacterial species at the genome-level, reflecting the common origin and specific features of target genome (Datta et al., 2020). The complete genome of HM-7 was compared with the other five closely releated *B. pumilus* strains (GR8, MTCC-B6033, TUAT1, C4, and SH-B11) to investigate the collinear relationship and orthologous distribution of genes. As a result, a total of 9 homologous regions were detected among the six genomes (Figure 5A). On the whole, a high degree of synteny was demonstrated among the six strains (regions with the same color). As for HM-7, three genomic regions with 107.3 kb (X), 153.2 kb (Y), and 36.8 kb (Z) were detected, which are obviously different from other *B. pumilus* strains.

Before dissecting the genes located in the specific-regions of HM-7, mobile genetic elements (MGEs) were analyzed, resulting in the identification of 21 genomic islands (GIs) including 5 putative prophages in the HM-7 genome (Figure 5B). Intriguingly, the X, Y, and Z specific-regions of HM-7 were overlapped with four prophages (177-100,490, 93,656-161,316, 2,508,533-2,591,517, and 3,920,319-3,951,028) (Figure 5B and Supplementary Table 6). The GC content of these specific-regions was relatively low in contrast to the average level of the genome (Figure 5B). In addition, three large repeat fragments were detected in the three regions (Figure 5C).

In total, we identified 424 strain-specific genes in these three specific-regions, accounting for 84.8% of the unique genes in HM-7 (Supplementary Table 7). Most of these genes were distributed in phage regions, suggesting that they were derived from phage-mediated lateral gene transfer (Figure 5C). In the case of pathogenic bacteria, strain-specific genes frequently encoded important virulence factors such as bacterial toxins (Fortier and Sekulovic, 2013). Herein, 19 candidate genes were concerned that might contribute to its pathogenesis (Table 2). Among them, the *uviB* and *pezT* genes were found in the region X that are associated with the export of bacteriocin and toxin, respectively. The *pseB* gene was predicted as VFDB factor ABZJ_00087, and encodes the UDP-N-acetylglucosamine- 4% 2C6-dehydratase, which is a sialic-acid-like sugar involved in flagellin modification and capsule formation (Hinderlich et al., 2013). It has been reported that phage tail genes seem to have developed dual functions and also serve as adhesion proteins for bacterial host attachment (Brüssow et al., 2004). Phage tail gene *pfbA* was predicted to encode the plasmin fibronectin-binding protein A, which binding to fibronectin and plasmin, working as virulence factors like adhesins/invasins for the LPXTG motif of the cell-anchoring sequence in Gram-positive bacteria (Yamaguchi et al., 2008). The *mepM* gene families were predicted to be the Murein DD-endopeptidase MepM, and functionally affected membrane invagination and cytokinesis (Babu et al., 2011). These candidate strain-specific genes located in the specific regions provided important information for parsing the pathogenesis of *B. pumilus* HM-7.

**TABLE 2:** The annotation of 19 specific-genes in HM-7 against the VFDB, PHI, and CAZYmes databases.

Specific gene	Predicted function	VFDB	PHI	CAZYmes
mepM_1	Murein DD-endopeptidase MepM	Adherence	reduced_virulence	GH23
pfbA_1	Plasmin and fibronectin-binding protein A	Adherence	–	GH0
mepM_2	Murein DD-endopeptidase MepM	Adherence	reduced_virulence	GH23
pfbA_2	Plasmin and fibronectin-binding protein A	Adherence	–	GH0
pseB_2	UDP-N-acetylglucosamine 4%2C6–dehydratase (inverting)	Immune modulation	–	–
HM–7_00154	DNA–binding protein HRL53	–	reduced_virulence	–
HM–7_04212	Cell wall-binding protein YocH	–	–	CBM50
HM–7_00001	Hypothetical protein	–	–	GH23
HM–7_00002	Hypothetical protein	–	–	GH23
HM–7_00003	Hypothetical protein	–	–	GH23
HM–7_00004	Hypothetical protein	–	–	GH23
HM–7_00008	Hypothetical protein	–	–	GH0
HM–7_00011	N-acetylmuramoyl-L-alanine amidase CwlA	–	–	CBM50
HM–7_00088	Cell wall-binding protein YocH	–	–	CBM50
HM–7_00187	Hypothetical protein	–	–	GH0
HM–7_00190	N-acetylmuramoyl-L-alanine amidase CwlA	–	–	CBM50
rhaS_3	HTH-type transcriptional activator RhaS	–	–	GH39
leuA_1	2-isopropylmalate synthase	–	reduced_virulence	–
TetD	Transposon Tn10 TetD protein	–	reduced_virulence	–

“–” indicates that no significant BLAST hit was found.

## Discussion

*B. pumilus* has been widely applied in industry and agriculture for its ability to produce substances with biocatalysis, antimicrobial, and plant growth promoting activities (Gutierrez-Manero et al., 2001; Saggese et al., 2018; Hayat et al., 2020). However, increasing studies have revealed some *B. pumilus* strains can cause opportunistic infections and elicit biosecurity concerns (Johnson et al., 2008; Logan, 2012; Peng et al., 2013; Song et al., 2018; Mazlan et al., 2019), although its pathogenesis is still vague. In recent years, genome sequencing approaches and genomic analysis strategies have provided powerful tools to facilitate researchers distinguishing pathogenic from non-pathogenic microbe and dissecting the underlying mechanisms of bacterial pathogens (Thomson et al., 2006; Panthee et al., 2021). In our previous report, *B. pumilus* HM-7 was identified as a pathogen of muskmelon, causing the bacterial soft rot (Song et al., 2018). Herein, we performed the genome assembly and comparative genomic analysis to clarify genomic features and determine the virulence genes for *B. pumilus* HM-7, offering valuable information for better utilization of *B. pumilus* strains in the future.

The ANI analysis with genomic information is commonly used for evaluating the genomic distance and establishing species boundaries, which overcomes the challenges caused by evolutionary mutation rates and HGT events (Konstantinidis and Tiedje, 2007). An accurate phylogenetic tree supports our understanding of the major transitions in evolution (Emms and Kelly, 2015; Kapli et al., 2020), the phylogenetic tree based on single-copies shared by all genomes is more accurate than a tree constructed from only the 16S rRNA gene (Hahnke et al., 2016). In our present study, core and pan-genome analysis of 27 *Bacillus* strains revealed extensive genetic diversity among *Bacillus* species and *B. pumilus* strains. Notably, *B. subtilis*, *B. licheniformis*, and *B. safensis*, are much closer to *B. pumilus* than other members of the *Bacillus* species (Supplementary Figures 2, 3), corroborating previous findings (Zhao et al., 2012; Fu et al., 2021). Additionally, tightly clustered SF-4, SAFR-032, and 150a, as well as the highly divergent of strain 145, were consistent with a previously reported study (Iqbal et al., 2021). As a result, we found that MTCC-B6033, TUAT1, C4, SH-B11, and GR8 were highly similar to HM-7 based on the evidence from ANI analysis and phylogenetic trees using single-copy genes. Intriguingly, HM-7 had a high proportion of strain-specific genes, which provided an important clue to investigate pathogenic genes. According to previous reports, MTCC-B6033, SH-B11, TUAT1, and C4 were identified as biocatalyst, antifungal, biofertilizer, and keratin-degrading (Villanueva et al., 2014; Fellahi et al., 2016; Zam et al., 2016; Okazaki et al., 2019). While GR8 and HM-7 strains exhibited pathogenic effects to ginger and muskmelon, respectively (Yuan and Gao, 2015; Song et al., 2018). It was supposed that GR8 and HM-7 might be more similar in terms of virulence.

Previous studies have been elaborated the capacities and processes of bacterial pathogens, including adherence to host tissues, invasion, modulation of host inflammatory responses, and secretion of toxic products (Ramarao and Sanchis, 2013; Do Vale et al., 2016; Gu et al., 2019). In this study, candidate pathogenic genes associated with adhesion, immune escape and toxicity were found and potentially responsible for the virulence of strain HM-7 (Supplementary Table 4). It was reported that biofilm formation and bacterial adherence of *B. amyloliquefaciens* were disrupted when the genes of collagen-like proteins (CLPs, ClpA, ClpB, ClpC, and ClpD) were inactivated (Zhao et al., 2015). Simultaneously, the flagellar biosynthesis-related genes (*fliD*, *fliE*, *flgD*, *flgE, flhA*) were expressed in pathogenicity-activated *Xanthomonas oryzae* pv. *oryzae* cells at the transcriptome and proteome levels (Kim et al., 2021). These adhesion-related genes were detected in HM-7 genome, which might play important roles during invasion in this bacterium. The bacterial pathogen needs to escape the immune assaults of host in various forms such as phagocytosis and complement-mediated killing. Previous reports revealed that anti-phagocytosis of immune escape were associated with capsules genes in *B. subtilis* and *B. anthracis* (Makino et al., 2002; Gu et al., 2019). Here, *cap* genes (*capB*, *capC*, and *capA*), cps genes (*cpsF*, *cpsG*, *cpsJ*), and the genes of LplA1, LspA, PanD, PanC, SodA, and SodB related to capsule biosynthesis in *Bacillus* species (Koehler, 2002; Gu et al., 2019), were also found in the HM-7 genome. Moreover, genes involved in nutritional/metabolic system, such as metal (e.g., zinc, iron and magnesium) uptake and adaptation, could partially explain the virulence of strain HM-7, since they play the crucial role in proliferation and pathogenicity of bacterial pathogens (Liu et al., 2022).

Until now, *B. pumilus* has caused a variety of symptoms on muskmelon, ginger, peach, pine, and Asian pear (Saleh et al., 1997; Kotan et al., 2006; Li et al., 2009; Peng et al., 2013; Kovaleva et al., 2015; Song et al., 2018), while the virulence factors involved in the pathogenic mechanism in *B. pumilus* still remain unclear. It is well known that bacterial soft rot results from the general disorganization of plant tissues following the degradation of the major component of primary cell walls (Bauer et al., 1994). Yuan and Gao (2015) confirmed that *B. pumilus* GR8 could cause ginger rhizome rot by producing plant cell wall-degrading enzymes to destroy ginger cells. It has also reported that hydrolytic enzymes could decompose plant cells and tissues without wounds by pathogenic *B. altitudinis* (Lemjiber et al., 2021). Phytopathogenic CAZymes, such as cellulases, pectinases, xylanases, and proteases, play a central role in plant cell wall degradation and facilitate bacterial colonization and nutrient acquisition (Cantarel et al., 2009; He et al., 2021). The candidate genes encoding CAZymes for vegetal tissue degradation may support the phytopathogenicity. The pectate lyase genes *pelA* and *pelD* in *B. subtilis* could degrade gum from the plant cell wall (Zou et al., 2013). The deletion of the endoglucanase gene *celA* or the pectinase gene *pelA1* in virulent strains of *Clavibacter michiganensis* can lead to significantly decreased pathogenicity and reduced canker symptom (Wang et al., 2022). The homologous genes *celY* and *pelA*, encoding glucanase and pectate trisaccharide lyase, respectively, were found in HM-7 genome.

Interestingly, we found that the presence of several genomic islands (GEIs) and prophages with large (>10 kb) integrative elements and repeat fragments in HM-7 genome, indicating that horizontal gene transfer (HGT) occurred during evolution. Virulence factors and pathogenicity determinants can spread via HGT through mobile genetic elements (MGEs) such as plasmids, bacteriophages, and genetic islands (GIs) (Dobrindt et al.,, 2004; Sobecky and Hazen, 2009). Phages play an important role in the evolution and virulence of bacterial pathogens for carrying key virulence factors (Brüssow et al., 2004). In HM-7 genome, varied GC-content around prophages suggested a phage-mediated gene transfer from a rare heterologous host differing in GC content. The molecular mechanisms underlying pathogenicity in bacteria might be related with the acquisition of new DNA sequences through HGT (Bartoli et al., 2016). Flagella are essential membrane structures that contribute to bacterial virulence and mediate the secretion of extracellular toxins (Sperandio et al., 2002). Toxins were capable of manipulating host cell functions and vital processes of living organisms to favor microbial infection (Do Vale et al., 2016). In this study, functional annotation of strain-specific genes in HM-7 led to the characterization of 19 candidate genes involved in flagella formation and toxin production, which may assist the activation of virulence in HM-7. Future work is necessary to reveal the functions of these candidate genes potentially involved in the pathogenicity of the bacterium associated with muskmelon fruit rot.

As previously reported, phages could effectively control the ginger rhizome rot caused by *B. pumilus* GR8 (Yuan et al., 2015; Yuan and Gao, 2016). Bacteriophages could be a promising antimicrobial approach as biocontrol agents against pathogens in animals, food products and plants (Goodridge and Bisha, 2011; Yuan et al., 2015; Gambino and Brøndsted, 2021). Considering that the CRISPR/Cas system was absent in HM-7, phage therapy could be a practical strategy for managing bacterial soft rot of muskmelon caused by strain HM-7 in the future.

In this study, the genome sequencing and comparative genomic analyses facilitated the determination of genomic features and virulence factors in the pathogenic strain *B. pumilus* HM-7. Our results explored to the current understanding of pathogenesis involved in the aspects of adhesion, invasion, intracellular survival, and evasion of host defenses, which would be applied in preventions of bacterial attack and infection in future. Admittedly, more efforts are required to validate the functions of these candidate genes and figure out the pathogenic models as well as involved physiological and metabolic processes.

## Data availability statement

The datasets presented in this study can be found in online repositories. The names of the repository/repositories and accession number(s) can be found below: https://www.ncbi.nlm.nih.gov/bioproject/PRJNA857747.

## Author contributions

QW and LXZ contributed to conception and design of the study. QW performed the bioinformatic data analyses and wrote the manuscript draft. LZ carried out collection and organized the data. YZ, HC, JS, ML, and RC revised the manuscript. All authors contributed to the article and approved the submitted version.

## Footnotes

1
http://ftp.ncbi.nlm.nih.gov/genomes

2
https://itol.embl.de/

3
https://phaster.ca/

## Supplementary material

Supplementary Figure 1 Gene annotation by GO, and KEGG for *B. pumilus* HM-7. **(A)** GO function classification of genes in HM-7. GO analysis was performed for three main categories: cellular components, molecular function, and biological processes. **(B)** The KEGG pathway classification of genes in HM-7 contains six groups: cellular processes, environmental, genetic, human diseases, metabolism, and organismal systems.

Supplementary Figure 2 Pan-core genome analysis of 27 strains of *Bacillus* species. **(A)** Gene distribution of 27 strains based on the gene presence-absence matrix generated from Roary. A purple box, green box and an orange box to represent the core gene, accessory genes and specific genes, respectively. The phylogenetic tree on the left represents the phylogenetic relationships among the strains that make up the corresponding pan-genome; **(B)** Flower petal plot of 27 strains. Pan-genome analysis of 27 strains yielded unique genes for each strain, the numbers of which are shown on each petal plot. The center Circos shows that the number of core gene obtained from 27 strains.

Supplementary Figure 3 Evolutionary relationships of 27 strains of *Bacillus* species. **(A)** Heatmap and dendrogram of average nucleotide identity among different strains. **(B)** Phylogenetic tree based on total single-copy orthologous.

Supplementary Table 1 Characteristics of the strains used in this study.

Supplementary Table 2 Genes presence and absence in six strains of *B. pumilus*. The presence of each CDS in the respective genome is listed below each labeled strain column. If a cell is blank the respective strain does not have the gene.

Supplementary Table 3 CAZymes categories identification in HM-7 using the CAZymes database.

Supplementary Table 4 Virulence-related factor identification in HM-7 using the VFDB database.

Supplementary Table 5 Gene identification in HM-7 using the PHI database.

Supplementary Table 6 Prophage regions identification in HM-7 using PHASTER.

Supplementary Table 7 Genes in three unique regions of HM-7.
