# Supplementary figures and images for: Comparative genomic analyses reveal genetic characteristics and pathogenic factors of Bacillus pumilus HM-7
Source: Front Microbiol. 2022 Nov 7;13:1008648. doi: 10.3389/fmicb.2022.1008648 (PMC9677121; doi:10.3389/fmicb.2022.1008648)

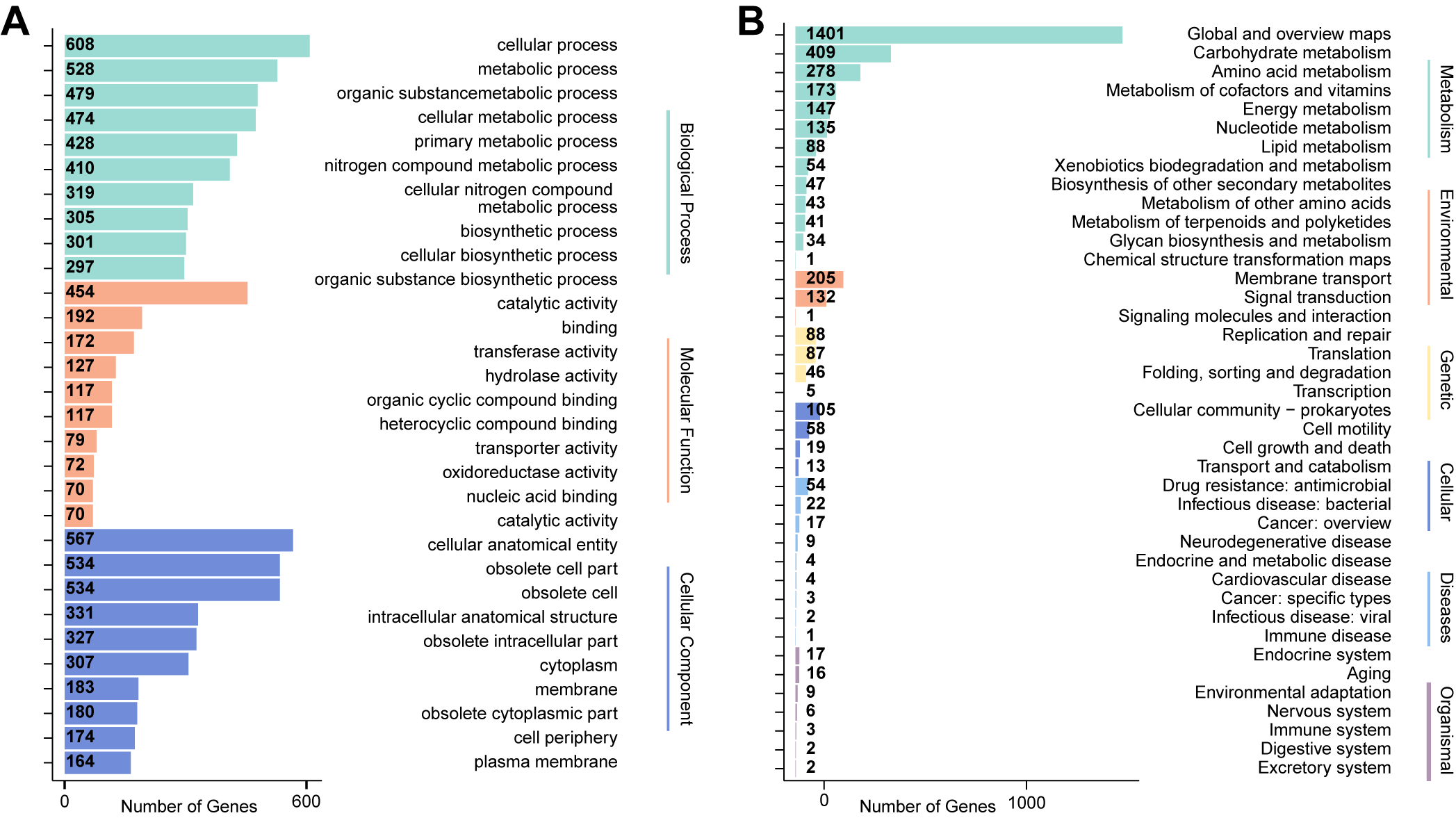

Supplement: Supplementary Figure 1 — Gene annotation by GO, and KEGG for B. pumilus HM-7. (A) GO function classification of genes in HM-7. GO analysis was performed for three main categories: cellular components, molecular function, and biological processes. (B) The KEGG pathway classification of genes in HM-7 contains six groups: cellular processes, environmental, genetic, human diseases, metabolism, and organismal systems. [file Image_1.tif]

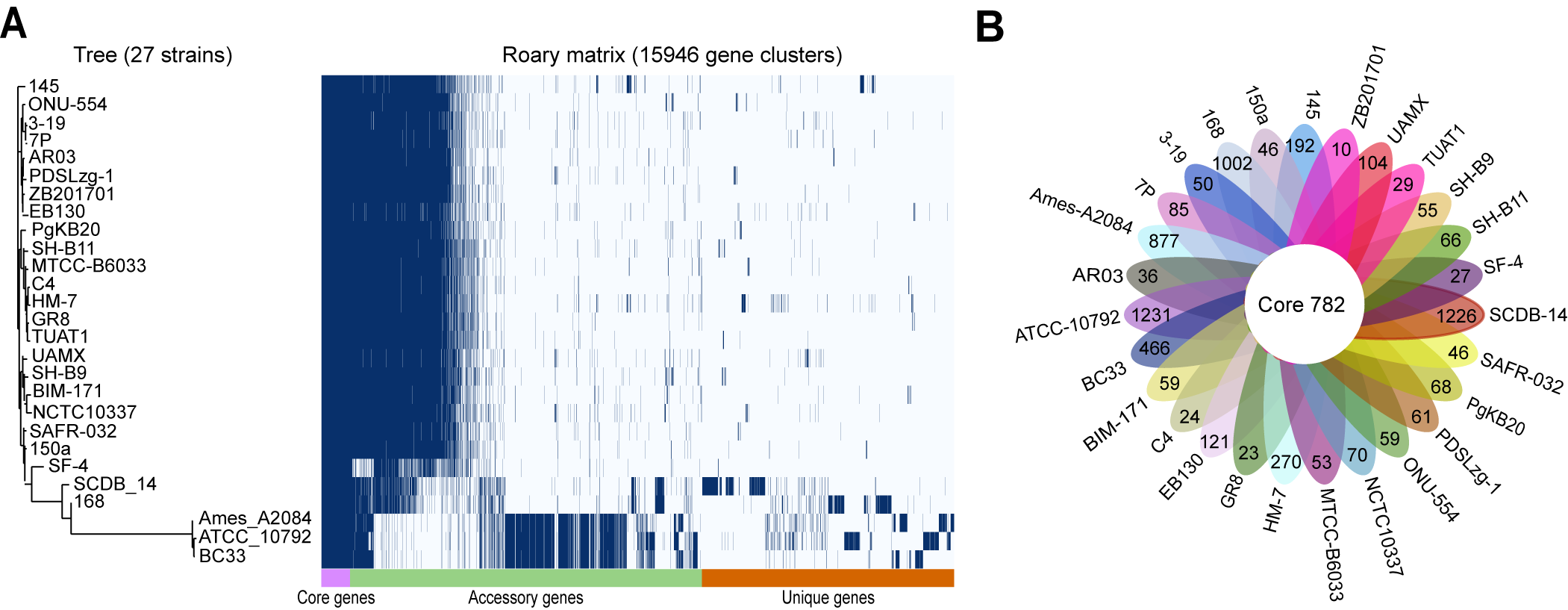

Supplement: Supplementary Figure 2 — Pan-core genome analysis of 27 strains of Bacillus species. (A) Gene distribution of 27 strains based on the gene presence-absence matrix generated from Roary. A purple box, green box and an orange box to represent the core gene, accessory genes and specific genes, respectively. The phylogenetic tree on the left represents the phylogenetic relationships among the strains that make up the corresponding pan-genome; (B) Flower petal plot of 27 strains. Pan-genome analysis of 27 strains yielded unique genes for each strain, the numbers of which are shown on each petal plot. The center Circos shows that the number of core gene obtained from 27 strains. [file Image_2.tif]

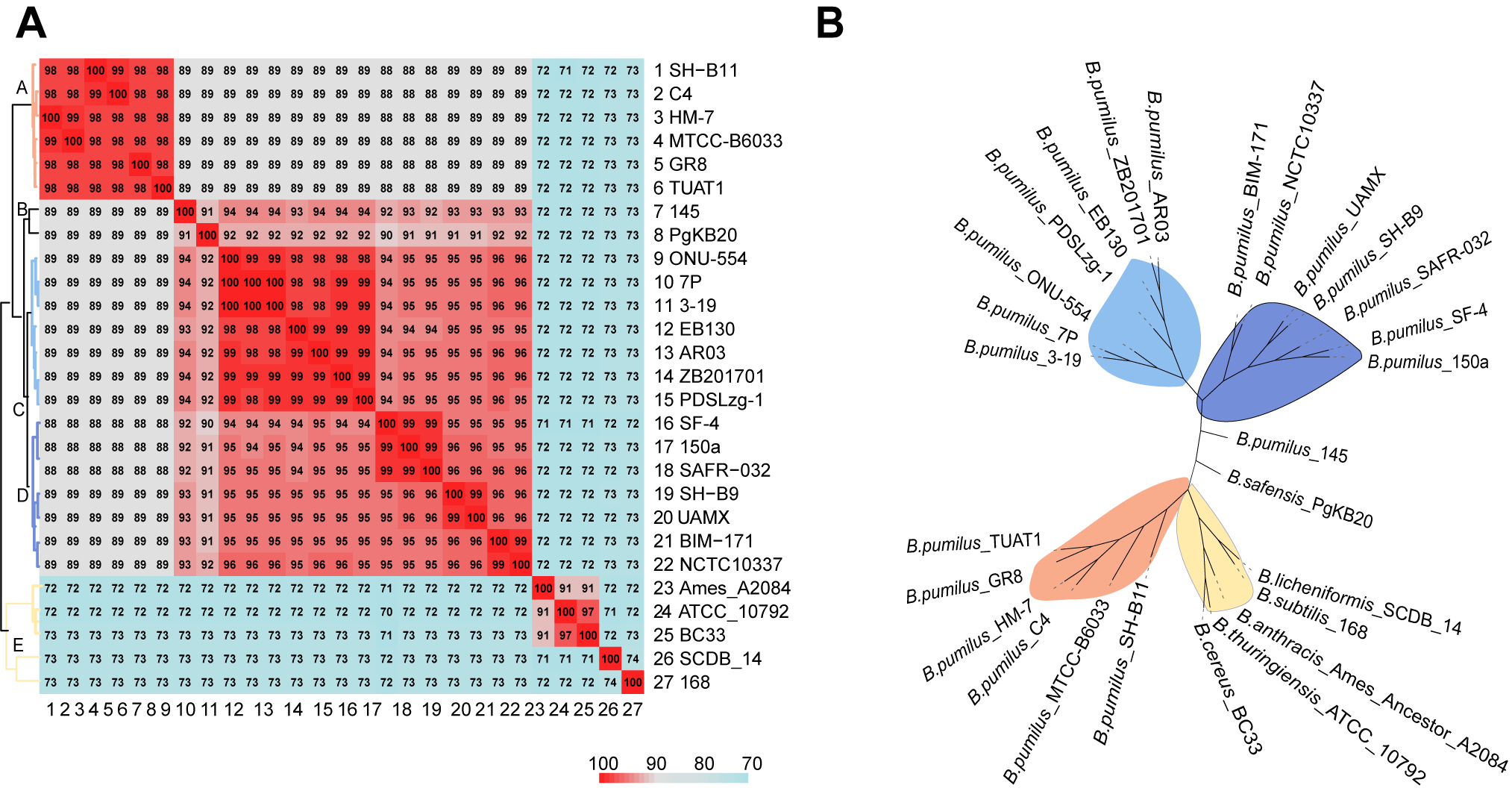

Supplement: Supplementary Figure 3 — Evolutionary relationships of 27 strains of Bacillus species. (A) Heatmap and dendrogram of average nucleotide identity among different strains. (B) Phylogenetic tree based on total single-copy orthologous. [file Image_3.tif]
